# Undifferentiated headache: broadening the approach to headache in children and adolescents, with supporting evidence from a nationwide school-based cross-sectional survey in Turkey

**DOI:** 10.1186/s10194-018-0847-1

**Published:** 2018-02-27

**Authors:** Christian Wöber, Çiçek Wöber-Bingöl, Derya Uluduz, Tuna Stefan Aslan, Uğur Uygunoglu, Ahmet Tüfekçi, Selen Ilhan Alp, Taşkın Duman, Fidan Sürgün, Gülser Karadaban Emir, Caner Feyzi Demir, Ferhat Balgetir, Yeliz Bahar Özdemir, Tanja Auer, Aksel Siva, Timothy J. Steiner

**Affiliations:** 1 Department of Neurology, Medical University of Vienna, Vienna, Austria; 2 Dr Gönül Bingöl-Dr Muammer Bingöl Çocuk ve Ergen Başağrısı Derneği, Istanbul, Turkey; 3 Neurology Department, Cerrahpaşa School of Medicine, Istanbul University, Istanbul, Turkey; 4 Neurology Department, Recep Tayyip Erdoğan University School of Medicine, Rize, Turkey; 5 Neurology Department, Namık Kemal University School of Medicine, Tekirdağ, Turkey; 6 Neurology Department, Mustafa Kemal University School of Medicine, Hatay, Turkey; 7 Neurology Department, Sıtkı Kocaman University School of Medicine, Mugla, Turkey; 8 Neurology Department, Fırat University School of Medicine, Elazığ, Turkey; 9 Department of Physical Medicine and Rehabilitation, Marmara University Medical School, Istanbuk, Turkey; 10 Department of Child and Adolescent Psychiatry, Medical University of Vienna, Vienna, Austria; 11 Department of Neuromedicine and Movement Science, NTNU Norwegian University of Science and Technology, Edvard Griegs Gate, Trondheim, Norway; 12 Division of Brain Sciences, Imperial College London, London, UK

**Keywords:** Headache, Undifferentiated headache, Migraine, Tension-type headache, Headache yesterday, Burden of headache, Quality of life, Children, Adolescents, Population-based study, Nationwide, Turkey, Global Campaign against Headache

## Abstract

**Background:**

Headache is a leading disabler in adults worldwide. In children and adolescents, the same may be true but the evidence is much poorer. It is notable that published epidemiological studies of these age groups have largely ignored headaches not fulfilling any specific set of ICHD criteria, although such headaches appear to be common. A new approach to these is needed: here we introduce, and investigate, a diagnostic category termed “undifferentiated headache” (UdH), defined in young people as recurrent mild-intensity headache of < 1 h’s duration.

**Methods:**

We conducted a nationwide cross-sectional survey in 31 schools in six regions of Turkey selected by mixed convenience-based and purposive modified cluster-sampling. A validated, standardised self-completed structured questionnaire was administered by a physician-investigator to entire classes of pupils aged 6–17 years.

**Results:**

Of the identified sample of 7889 pupils, 7088 (89.8%) participated. The 1-year prevalence of UdH was 29.2%, of migraine (definite and probable) 26.7%, and of tension-type headache (TTH) (definite and probable) 12.9%. UdH differed with respect to almost all headache features and associated symptoms from both migraine and TTH. Burden of headache and use of acute medication were lower in UdH than in migraine and TTH. Headache yesterday was less common in UdH than migraine (OR 0.32; 95% CI 0.28–0.37) and TTH (OR 0.64; 95% CI 0.56–0.77). Quality of life (QoL) was better in UdH (33.6 ± 5.2) than in migraine (30.3 ± 5.6; *p* < 0.001) and TTH (32.4 ± 5.3; *p* < 0.001), but worse than in pupils without headache (35.7 ± 4.7; *p* < 0.001).

**Conclusions:**

This large nationwide study in Turkey of pupils aged 6–17 years has shown that many children and adolescents have a headache type that does not conform to existing accepted diagnostic criteria. This new diagnostic category of presumably still-evolving headache (*undifferentiated headache*) is common. UdH differs in almost all measurable respects from both migraine and TTH. Although characterised by mild headaches lasting < 1 h, UdH is associated with significant adverse impact on QoL. Longitudinal cohort studies are needed to evaluate the prognosis of UdH but, meanwhile, recognition of UdH and its distinction from migraine and TTH has implications for epidemiological studies, public-health policy and routine clinical practice.

## Background

In the Global Burden of Disease Study 2010 (GBD2010) [1], tension-type headache (TTH) and migraine were revealed as second and third most prevalent disorders in the world. In GBD2013, headache disorders collectively were third among the leading causes of disability [2]. In GBD2016, migraine came top – the single most disabling disorder – in the age group 15–49 years [3].

The data underpinning these statistics were derived in the main from studies in adults aged 18–65 years. In children (6–11 years) and adolescents (12–17 years), the prevalence of headache disorders is not well established and the burdens attributable to them are poorly characterised. Multiple factors explain this. First, there are relatively few published epidemiological studies [4] and, second, most of these have been conducted in middle- or high-income Western countries, leaving very large geographical gaps [4]. Third, substantial methodological differences between them limit their comparability. Nonetheless, extrapolations to these age groups in these global surveys put migraine among the top 10 global causes of years lived with disability (YLDs) in the 50 most populous countries [5].

An additional factor arises from the operational diagnostic criteria set out in the universally accepted International Classification of Headache Disorders (ICHD), now in its third edition (ICHD-3) [6]. While ICHD recognises > 200 headache disorders, only migraine and TTH among the primary headache disorders have public-health importance [1–4]. Medication-overuse headache (MOH), with an adult mean global prevalence of 1.5–2% [7], also contributes substantially to global disability [2, 3]. The ICHD diagnostic criteria for migraine in adults specify recurrent moderate-to-severe headache of 4–72 h’ duration, with a range of specific characteristics (unilaterality, pulsating quality, aggravation by physical activity) and accompanying symptoms (photophobia *and* phonophobia; nausea and/or vomiting) [6]. In children it is noted that the headache may be of shorter duration (2–72 h). The criteria for TTH specify mild-to-moderate headache lasting from 30 min to 7 days, with neither the specific characteristics nor the accompanying symptoms of migraine [6]. In our pilot school-based prevalence survey conducted in Turkey and Austria, mild headache of < 1 h’s duration was reported by a large proportion (37.2%) of participants aged 6–17 years, often with migraine-like features [8].

Such headache could not be given a definite diagnosis of migraine or, when there were migraine-like features, of TTH. Furthermore, although ICHD criteria for TTH have, from the first edition onwards [9], specified a lower duration limit of 30 min, we questioned whether this disorder existed in a form in which headache was *typically* of < 1 h’s duration. Therefore, in the context of epidemiological studies, we found ourselves in doubt as to how this common presentation of headache should be labelled.

The literature offered no guidance. In the most recent survey [10] and a Medline search (using the terms “migraine”, “tension-type headache”, “children”, “adolescents”, “prevalence” and “epidemiology” without language restrictions), we found 59 relevant studies. The majority (54: 91.5%) reported only selected diagnoses (most often definite migraine), and, crucially, did not specify the proportion of unclassifiable headaches. However, among the five studies that did report them [11–15], unclassifiable headaches were common, with an average prevalence of almost 20%. In other words, the problem we encountered had been recognised previously – but ignored.

In the present study, we investigate whether a new approach is required in children and adolescents, recognising that the characteristics of adult migraine (and perhaps TTH) may be undeveloped in 6–17 year-olds. We introduce an additional diagnostic category defined by headache duration of < 1 h and mild intensity, which we have termed “undifferentiated headache” (UdH). In a nationwide school-based epidemiological survey in Turkey, we compare the clinical features of UdH with those of migraine and TTH. We also compare the burdens attributable to each, including impact on quality of life (QoL). Our purpose is to establish whether or not UdH should be regarded for epidemiological and perhaps clinical purposes as distinct from migraine and TTH.

The survey was conducted under the auspices of the Global Campaign against Headache [16], which is directed by the UK-registered non-governmental organisation *Lifting The Burden* (LTB) in official relations with the World Health Organization [17], and of Dr. Gönül Bingöl-Dr Muammer Bingöl Çocuk ve Ergen Başağrısı Derneği.

## Methods

### Ethics and approvals

The protocol was approved by the ethics committee of the co-ordinating centre (Istanbul University no. 83045809/604/02–12,472). Since the study involved multiple schools nationwide, approval was also obtained from the Ministry of National Education. Copies of these approvals were sent to the school managers to obtain permissions to visit.

Informed consent, in terms agreed within these approvals, was given by or on behalf of each child or adolescent before his or her participation.

Data were collected anonymously and managed in accordance with data-protection legislation.

### Study design

The cross-sectional survey applied the methods developed and tested in pilot studies performed in Istanbul and Vienna [8]. Conducted nationwide in selected schools, it employed a self-completed structured questionnaire administered to entire classes. It was performed within one academic term, avoiding examination periods, between April 4th and June 13th 2014.

### Sampling and subjects

We used mixed convenience-based and purposive modified cluster-sampling. The procedure was multistage: (1) selection of sites in each of six regions of Turkey, reflecting the country’s geographical and socioeconomic diversity; (2) identification by the local investigators of three or more schools within each site to reflect, as far as possible, its socioeconomic and urban/rural diversity; (3) selection of classes in each school across the age ranges 6–11 and/or 12–17 years (randomly, when there were more than one for any particular year); and (4) inclusion of all eligible children in the classes.

The survey sites were six cities and their environs: Elâzığ (eastern Anatolia, in uppermost Euphrates valley); Hatay (southern Turkey, close to the Syrian border) Istanbul (north-western Turkey, astride the Bosphorus); Muğla (south-western Turkey, close to the Aegean coast); Rize (north-eastern Turkey, on the Black Sea coast), and Tekirdağ (European side, on the northern coast of the Sea of Marmara).

Selected schools were invited to participate. Within each class, all eligible pupils were included except for those who refused, were unable to take part for any reason, or were absent from school on the interview day.

### Questionnaires

The questionnaire was an adaptation of the HARDSHIP questionnaire [18] in separate versions for children and adolescents. These were translated into Turkish language in accordance with LTB’s translation protocol for hybrid documents to capture exact meaning rather than simple linguistic equivalence [19]. Each version had been evaluated with satisfactory results in the pilot studies [8], requiring < 30 min to complete. The questionnaires were administered to the pupils in class by trained medical residents, introduced by their teacher (the mediator). The mediator took steps to prevent any participant copying the responses of another.

Questions were included on demographics, headache occurrence, ICHD-3 beta diagnostic criteria (no different, with respect to migraine and TTH, from those of ICHD-3 [6]), burden attributable to headache, and QoL. A short questionnaire completed by the mediator recorded details about the school and its local environment. A further very brief questionnaire also completed by the mediator documented non-participation. Both these questionnaires have been published previously [8].

### Headache diagnosis

Diagnoses were made using the HARDSHIP algorithm [18], but we modified the process to include UdH. First, we applied the criteria for UdH (duration < 1 h, intensity mild). Among remaining participants, we separated those reporting headache on ≥15 days/month, diagnosing probable MOH (pMOH) when acute medication was reportedly used on ≥15 days/month or, otherwise, “other headache on ≥15 days/month” (these cases were not included in this analysis). To those still remaining (with episodic headache of duration ≥1 h and/or intensity moderate or severe), we applied the criteria, in order, for definite migraine, definite TTH, probable migraine and finally probable TTH [20]; criterion B for TTH [6] was modified by raising the lower limit of duration to 1 h.

### Statistics and analyses

Recommendations on sample-size calculation for headache *prevalence* suggest limited gain from samples of > 2000 participants [20]. For *burden*, larger numbers may be more informative, but there is no good basis for power calculation. We aimed for 4000 evaluable participants per age range, spread as evenly overall as possible by recruiting across the age groups in each school.

Completed questionnaires were conveyed to and held securely at Istanbul University, where data were transferred into a secure electronic database.

Many questions required “yes/no” responses. Headache duration was reported in hours and intensity as “not bad”, “quite bad” or “very bad”, which we equated to mild, moderate and severe. Frequencies were reported in days per week or 4 weeks. Emotional impact and QoL questions required selection from the response options “never”, “sometimes”, “often” and “always” in the preceding 4 weeks; these were scored 1–4, and summed to generate impact (potential range 6–24; high being adverse) and QoL scores (12–48; low being adverse).

For descriptive analyses we generated proportions (%) and, where appropriate, means and standard deviations (SDs). In comparative analyses, we applied Student’s t-test to parametric variables, Kruskal Wallis test to nonparametric continuous variables and chi-squared test to categorical variables. We used bivariate analysis to estimate odds ratios (ORs) with 95% confidence intervals (CIs).

We used IBM SPSS Statistics, Version 23, for all calculations. To adjust for multiple comparisons, Bonferroni correction was performed online [21] following the suggestions of Sankoh et al. [22]. In the case of correlated outcome variables, Bonferroni correction is too conservative; the variables in our study were highly correlated with each other: calculating Pearson correlation coefficients we found a statistically significant correlation in 227 of 253 correlations (90%). Therefore, the *p*-value for significance required adjustment only to *p* < 0.03.

## Results

### Study population

We invited 31 schools (2–10 [mean 5.2 ± 2.6] per site), 288 classes (2–32 [mean 9.4 ± 6.6] per school) and 7889 pupils (3–167 [27.4 ± 16.1] per class). None of the invited schools or classes declined to participate. Of the 7889 invited pupils, 7088 (89.8%) participated and 7068 (99.7%; 89.6% overall) completed questionnaires were evaluable (2551 children, 4517 adolescents). We did not achieve the intended *N* = 4000 children because of skewed age-distributions within the schools. Mean participation proportion of pupils per class was 90.8 ± 12.1% with no gender difference (girls 91.1 ± 14.4%; boys 90.5 ± 13.6%) and similar across age-groups (lowest [86.9 ± 17.7%] in pupils aged 15–16 and highest [95.1 ± 5.6%] in those aged 11–12 years). Pupil numbers per site ranged from 578 to 1855, and per age group from 290 (6–7 years) to 1146 (13–14 years).

Reasons for non-participation in the 10.2% were inadequately established despite the mediator’s questionnaire for this purpose, and cannot be reported.

### Schools

The proportions of schools in urban, semiurban and rural regions were 42.4%, 43.1% and 14.6% (compared with national urban and rural proportions extrapolated from 2011 data of about 77% and 23% [23]). The proportions in high-income, upper middle-, lower middle- and low-income districts were 2.8%, 27.1%, 68.4% and 1.7%.

### Headache prevalence

Headache during the previous year was reported by 73.7% of participants. We found the following 1-year prevalences: UdH 29.2%; migraine 26.7% (7.3% definite, 19.4% probable); TTH 12.9% (definite 6.7%, probable 6.2%); pMOH 0.9%; other headache on ≥15 days/month 3.4%; unclassifiable headache 0.5%. With concern for possible interest bias among responders, we also calculated prevalences with reference to the target sample of 7889 pupils: UdH 26.2%; migraine 23.9% (definite 6.5%, probable 17.4%); TTH 11.6% (definite 6.2%, probable 5.4%); pMOH 0.8%; other headache on ≥15 days/month 3.0%; unclassifiable headache 0.5%.

Further analyses focus on UdH, migraine and TTH, combining definite and probable cases of the latter two as recommended in guidelines [20].

### Headache characteristics and associated symptoms

Classifying UdH according to frequency analogously to TTH, we recorded infrequent episodic UdH in 26.7%, frequent episodic UdH in 72.0% and chronic UdH in 1.3% of cases.

UdH differed with respect to almost all headache features and associated symptoms from both migraine and TTH (Table 1). Headache days both in the previous week and in the previous 4 weeks were fewer in UdH than in migraine and TTH; in keeping with this, headache yesterday was less common in UdH than in migraine and TTH (Table 2). Intensity of headache yesterday was lower in UdH than in migraine and TTH. In contrast to the general rating of headache intensity as mild in UdH, 16.3% of pupils with the diagnosis of UdH rated headache yesterday as moderate or severe (Table 2).

**Table 1:** Headache features in undifferentiated headache, migraine and tension-type headache

	Undifferentiated headache		Migraine^a^		Tension-type headache^a^		Undifferentiated headache vs migraine^a^		Undifferentiated headache vs tension-type headache^a^	
	*N* = 2066		*N* = 1888		*N* = 911		OR [95% CI]	*p* ^b^	OR [95% CI]	*p* ^b^
	*n*	%	*n*	%	*n*	%				
Headache in previous week	2036		1864		899					
No	949	46.6	417	22.4	322	35.8	reference		reference	
≥ 1 day	1087	53.4	1447	77.6	577	64.2	0.33 [0.29–0.38]	< 0.001	0.64 [0.54–0.75]	< 0.001
Headache in previous 4 weeks	2050		1880		898					
No	548	26.7	207	11.0	161	17.9	reference		reference	
≥ 1 day	1502	73.3	1673	89.0	737	82.1	0.34 [0.29–0.40]	< 0.001	0.60 [0.49–0.73]	< 0.001
Duration (hr)	2066		1877		910					
< 1	2066	100	317	16.9	145	15.9	NA	NA	NA	NA
1–2	0	0	989	52.7	657	7.2				
> 2 and < 4	0	0	370	19.7	77	8.5				
≥ 4	0	0	201	10.7	31	3.4				
Intensity	2066		1877		907					
Mild	2066	100	537	28.5	516	56.9	NA	NA	NA	NA
Moderate	0	0	1046	55.4	319	35.2				
Severe	0	0	304	16.1	72	7.9				
Quality	2044		1885		908					
Pulsating	1437	70.3	1478	78.4	555	61.1	reference		reference	
Pressing	607	29.7	407	21.6	353	38.9	1.5 [1.3–1.8]	< 0.001	0.66 [0.56–0.78]	< 0.001
Localisation	2048		1887		906					
Unilateral	645	31.5	463	24.5	158	17.4	0.90 [0.76–1.07]	NS	1.7 [1.4–2.1]	< 0.001
Middle	652	31.8	421	22.3	269	29.7	reference		reference	
Bilateral	751	36.7	1003	53.2	479	52.9	0.48 [0.41–0.56]	< 0.001	0.65 [0.54–0.78]	< 0.001
Aggravation by physical activity	2059		1886		910					
No	1356	65.9	596	31.6	509	55.9	reference		reference	
Yes	703	34.1	1290	68.4	401	44.1	0.24 [0.21–0.27]	< 0.001	0.66 [0.56–0.77]	< 0.001
Avoidance of physical activity	2060		1888		911					
No	1009	49.0	319	16.9	408	44.8	reference		reference	
Yes	1051	51.0	1569	83.1	503	55.2	0.21 [0.18–0.25]	< 0.001	0.85 [0.72–0.99]	0.04
Nausea	2053		1887		909					
No	1381	67.3	578	30.6	751	82.6	reference		reference	
Yes	672	32.7	1309	69.4	158	17.4	0.22 [0.19–0.25]	< 0.001	2.3 [1.9–2.8]	< 0.001
Vomiting	2054		1887		902					
No	1842	89.7	1364	72.3	845	93.7	reference		reference	
Yes	212	10.3	523	27.7	57	6.3	0.30 [0.25–0.36]	< 0.001	1.7 [1.3–2.3]	< 0.001
Photophobia	2058		1883		908					
No	1385	67.3	622	33.0	752	82.8	reference		reference	
Yes	673	32.7	1261	67.0	156	17.2	0.24 [0.21–0.24]	< 0.001	2.3 [1.9–2.9]	< 0.001
Phonophobia	2062		1887		909					
No	257	12.5	72	3.8	125	13.8	reference		reference	
Yes	1805	87.5	1815	96.2	784	86.2	0.28 [0.21–0.37]	< 0.001	1.1 [0.89–1.4]	0.3

^a^Including definite and probable; ^b^chi-squared test

**Table 2:** Headache yesterday in undifferentiated headache, migraine and tension-type headache

Headache yesterday	Undifferentiated headache		Migraine^a^		Tension-type headache^a^		Undifferentiated headache vs migraine^a^		Undifferentiated headache vs tension-type headache^a^	
	*N* = 2066		*N* = 1888		*N* = 911		OR [95% CI]	*p* ^b^	OR [95% CI]	*p* ^b^
	*n*	%	*n*	%	*n*	%				
Present	2027		1872		895					
No	1624	80.1	1053	56.3	645	72.1	reference			
Yes	403	19.9	819	43.7	250	29.9	0.32 [0.28–0.37]	< 0.001	0.64 [0.56–0.77]	< 0.001
Intensity	403		819		250					
Mild	328	83.7	408	50.6	151	62.7	3.9 [2.8–5.4]	< 0.001	2.8 [1.9–4.2]	< 0.001
Moderate	55	14.0	265	32.9	72	29.9	reference		reference	
Severe	9	2.3	133	1.5	18	7.4	0.33 [0.16–0.68]	0.002	0.66 [0.27–1.6]	0.3
Loss of lessons at school	403		819		250					
No	360	90.2	693	87.3	217	89.7	reference		reference	
Yes	39	9.8	101	12.7	25	10.3	0.74 [0.50–1.1]	0.1	0.94 [0.55–1.6]	0.8

^a^Including definite and probable; ^b^chi-squared test

### ICHD criteria

Criterion B (duration < 1 h) was predefined for UdH. Criterion C for migraine without aura was fulfilled by 57.4% of UdH cases and criterion C for episodic TTH by 86.1%***.*** Criterion D for each was fulfilled by 55.1% and 44.9% of UdH cases. The proportions of pupils with UdH fulfilling none, one or two of criteria C and D for migraine without aura were 20.7%, 45.7% and 33.5%. The proportions who fulfilled none, one or two of criteria C and D for episodic TTH were 8.7%, 52.0% and 39.3%.

### Burden of headache

The proportions of pupils who missed school lessons because of headache yesterday did not differ between the three groups (Table 2), but impact on school attendance and leisure-time activities in the preceding 4 weeks was lower in UdH than in migraine and TTH. UdH was less likely than migraine, but not TTH, to cause a parent to leave work (Table 3).

**Table 3:** Burden of undifferentiated headache, migraine and tension-type headache (preceding 4 weeks)

Burden measure	Undifferentiated headache		Migraine^a^		Tension-type headache^a^		Undifferentiated headache vs migraine^a^		Undifferentiated headache vs tension-type headache^a^	
	*N* = 2066		*N* = 1888		*N* = 911		OR [95% CI]	*p* ^b^	OR [95% CI]	*p* ^b^
	*n*	%	*n*	%	*n*	%				
School absence	2046		1866		900					
No	1792	87.6	1439	76.2	753	83.7	reference		reference	
≥ 1 day	254	12.4	427	23.8	147	16.3	0.48 [0.40–0.57]	< 0.001	0.73 [0.59–0.91]	0.004
School left early	1997		1818		884					
No	1819	91.1	1479	81.4	766	86.7	reference		reference	
≥ 1 day	17	8.9	339	18.6	118	13.3	0.43 [0.35–0.52]	< 0.001	0.64 [0.50–0.81]	< 0.001
Impact on activity	2015		1800		887					
No	1261	62.6	582	32.3	459	51.7	reference		reference	
≥ 1 day	754	37.4	1218	67.7	428	48.3	0.29 [0.25–0.33]	< 0.001	0.64 [0.55–0.75]	< 0.001
Parent took work leave	2044		1880		902					
No	1906	93.2	1607	85.5	832	92.2	reference		reference	
≥ 1 Day	138	6.8	273	14.5	70	7.8	0.43 [0.34–0.53]	< 0.001	0.86 [0.64–1.2]	0.3

^a^Including definite and probable; ^b^chi-squared test

UdH imposed less emotional impact (summed impact score 11.3 ± 2.7) than migraine (13.6 ± 3.0; *p* < 0.001) and TTH (11.9 ± 2.7; *p* < 0.001).

### Quality of life

UdH adversely affected QoL (summed QoL score 33.6 ± 5.2 versus 35.7 ± 4.7 [*p* < 0.001] in pupils without headache), but to a lesser extent than migraine (30.3 ± 5.6; *p* < 0.001) and TTH (32.4 ± 5.3; *p* < 0.001).

### Use of acute medication

Pupils with UdH took acute medication on fewer days than those with migraine or TTH. Nevertheless, large proportions of those with UdH reported use of acute medication in the previous week (38.9%) and 4 weeks (43.2%) (Table 4).

**Table 4:** Use of acute medication in undifferentiated headache, migraine and tension-type headache

Use of acute medication	Undifferentiated headache		Migraine^a^		Tension-type headache^a^		Undifferentiated headache vs migraine^a^		Undifferentiated headache vs tension-type headache^a^	
	*N* = 2066		*N* = 1888		*N* = 911		OR [95% CI]	*p* ^b^	OR [95% CI]	*p* ^b^
	*n*	%	*n*	%	*n*	%				
Previous week	1074		1390		566					
None	656	61.1	555	39.9	279	49.3	reference		reference	
≥ 1 day	418	38.9	835	60.1	2873	50.7	0.42 [0.36–0.50]	< 0.001	0.62 [0.50–0.76]	< 0.001
Previous 4 weeks	1490		1620		725					
None	846	56.8	513	31.7	324	44.7	reference		reference	
≥ 1 day	644	43.2	1107	68.3	401	55.3	0.35 [0.31–0.41]	< 0.001	0.62 [0.51–0.76]	< 0.001

^a^Including definite and probable; ^b^chi-squared test

## Discussion

In this large nationwide sample of pupils aged 6–17 years in Turkey, we identified a headache type which we termed “undifferentiated headache” (UdH). UdH differed measurably with respect to almost all headache features and associated symptoms from both migraine and TTH. Although characterised by mild headaches lasting < 1 h, UdH was associated with significant burden, including adverse impact on QoL. Crucially, UdH was common, affecting almost 30% of the pupils.

It is arguable that UdH meeting two criteria of migraine without aura (as did one third of the cases diagnosed as UdH) might better be classified as probable migraine, and UdH meeting two criteria of TTH (almost 40% of cases) might better be classified as probable TTH. Longitudinal studies suggest, however, that a considerable proportion (8.3–71%) of children and adolescents with migraine evolve to TTH or vice versa [24]. In a 7-month follow-up study, diagnostic stability was higher in definite migraine (76.7%) and definite TTH (57.1%) than in probable migraine (44.7%) or probable TTH (43.3%) [25]. The findings of our study reflect this, and support the concept of UdH as a more appropriate diagnosis among these age groups than probable migraine or probable TTH.

With respect to headache duration, in order to accommodate the diagnosis of UdH, we necessarily modified the ICHD criteria for TTH, raising the minimum duration from 30 min to 1 h. As we noted earlier, we question whether TTH – in children or adults – really exists in a form in which headache of < 1 h’s duration is *typical*. Although epidemiological studies indicate that it does, we suggest that such reported occurrences are the consequence of treatment: many participants may not have experienced untreated attacks for many years [20].

In previous epidemiological studies of headache in children and adolescents, we were interested in whether authors classified headache in every participant who screened positively for headache or, instead, selectively reported cases fulfilling any specific set of ICHD diagnostic criteria. To clarify this, we re-reviewed all 50 studies included in the most recent review [10] and nine later studies focusing on TTH identified in a literature search up to April 2016 [11, 26–33]. Only five of the 59 studies reliably reported headache that was not classifiable by ICHD criteria [11–15], with prevalences of 2.9–35.5% (mean 18.3 ± 14.1) but with participation proportions ranging between 54.0% and 98.3% (mean 76.2 ± 19.8). In summary, past studies have generally ignored headaches not fulfilling ICHD criteria for migraine or TTH, which nonetheless appear to be common. There is no evidence from the publications that they met our criteria for UdH, but we offer this as a likely possibility.

There are implications in our proposal both for ICHD and for future epidemiological studies in these age groups, which may need to recognise UdH. There are also clinical implications if UdH, while clearly distinct from migraine, is in fact a precursor or immature form of it. We should emphasise that we propose UdH at this stage as a new diagnostic category, *not* as a new entity. Future research is needed, both to characterise UdH more comprehensively and, especially, to evaluate its prognosis. Only longitudinal cohort studies, ideally into adulthood and perhaps subdividing UdH according to the associated symptoms, can achieve the latter.

### Strengths and limitations

The strengths of this study lay in the large numbers of schools, classes and participants, the wide age range, the even distribution of girls and boys, the participants’ geographical and socio-economic diversity, the use of a validated questionnaire presented to the pupils in class by a physician and a high participation proportion.

The study was limited by its convenience-based rather than random sampling. Schools from urban regions (41%) were underrepresented (the national urban proportion is 77% [23]) and those from lower-middle income regions (68%) were overrepresented (Turkey is an upper-middle income country [34]). With respect to the features of UdH, we believe these deviations were not important. We relied on questionnaires, and, as in all such studies, were dependent on participants’ recall and accurate reporting. This is imperfect methodology [20], but it is not easily improved. While young children in particular may report imprecisely, enquiry of parents not only requires greatly increased resources but also, since it involves second-hand reporting, may not be more reliable. Lastly, our study refers to pupils from Turkey, and UdH needs to be assessed in other countries.

## Conclusion

Undifferentiated headache characterised by mild headaches lasting < 1 h is common in children and adolescents. It differs measurably with respect to almost all headache features and associated symptoms from both migraine and TTH and has significant adverse impact on QoL. This new diagnostic category in these age groups offers an alternative to jamming an evolving headache disorder that is neither clearly migraine nor clearly TTH into either of these diagnoses. Future longitudinal studies will show whether UdH represents those headaches that are in a shifting state between migraine and TTH before maturing by adulthood into one or the other. Differentiating UdH from migraine and TTH therefore has implications not only for epidemiology but also in routine clinical practice, since patients diagnosed with UdH call for closer follow-up with regard to their headache characteristics and associated symptoms, and a different therapeutic approach.
